# New Echocardiographic Findings Correlate with Intramyocardial Inflammation in Endomyocardial Biopsies of Patients with Acute Myocarditis and Inflammatory Cardiomyopathy

**DOI:** 10.1155/2013/875420

**Published:** 2013-03-20

**Authors:** Felicitas Escher, Mario Kasner, Uwe Kühl, Johannes Heymer, Ursula Wilkenshoff, Carsten Tschöpe, Heinz-Peter Schultheiss

**Affiliations:** ^1^Department of Cardiology and Pneumology, Charité-University Medicine Berlin, Campus Benjamin Franklin, 12203 Berlin, Germany; ^2^Berlin-Brandenburg Centre for Regenerative Therapies (BCRT), Charité, 13353 Berlin, Germany; ^3^DZHK (German Centre for Cardiovascular Research), Partner Site Berlin, Charité, 13347 Berlin, Germany

## Abstract

*Background*. The diagnosis of acute myocarditis (AMC) and inflammatory cardiomyopathy (DCMi) can be difficult. Speckle tracking echocardiography with accurate assessments of regional contractility could have an outstanding importance for the diagnosis. *Methods and Results*. *N* = 25 patients with clinically diagnosed AMC who underwent endomyocardial biopsies (EMBs) were studied prospectively. Speckle tracking imaging was examined at the beginning and during a mean follow-up period of 6.2 months. In the acute phase patients had markedly decreased left ventricular (LV) systolic function (mean LV ejection fraction (LVEF) 40.4 ± 10.3%). At follow-up in *n* = 8 patients, inflammation persists, correlating with a significantly reduced fractional shortening (FS, 21.5 ± 6.0%) in contrast to those without inflammation in EMB (FS 32.1 ± 7.1%, *P* < 0.05). All AMC patients showed a reduction in global systolic longitudinal strain (LS, −8.36 ± −3.47%) and strain rate (LSR, 0.53 ± 0.29 1/s). At follow-up, LS and LRS were significantly lower in patients with inflammation, in contrast to patients without inflammation (−9.4 ± 1.4 versus −16.8 ± 2.0%, *P* < 0.0001; 0.78 ± 0.4 versus 1.3 ± 0.3 1/s). LSR and LS correlate significantly with lymphocytic infiltrates (for CD3 *r* = 0.7, *P* < 0.0001, and LFA-1 *r* = 0.8, *P* < 0.0001). *Conclusion*. Speckle tracking echocardiography is a useful adjunctive assisting tool for evaluation over the course of intramyocardial inflammation in patients with AMC and DCMi.

## 1. Introduction

Acute myocarditis (AMC) is a serious disorder, whereby the clinical course and presentation are highly variable [1, 2]. Acute “infarct-like” changes to the ECG, a positive troponin T measurement, and a finding of edema, in patients with clinically suspected myocarditis indicates nonspecifically, virus-associated or inflammatory cell-associated injury to the myocardium. Both complete recovery of left ventricular (LV) function and progression to LV dysfunction have been described. Subsequently, dilated cardiomyopathy (DCM) may result from chronic inflammatory activation due to an inadequate immune response [3].

To make a specific diagnosis on which to base causative treatment, it is important to take endomyocardial biopsies (EMBs) [4–6]. The immunohistochemical characterization of infiltrates compared with LV dysfunction has led to a new entity of secondary cardiomyopathies acknowledged by the WHO, the inflammatory cardiomyopathies (DCMi). Ongoing viral persistence and intramyocardial inflammation diagnosed in EMBs have been associated with an adverse prognosis in DCM with an increased risk of cardiac death or the need or cardiac transplantation [7–11]. EMB-based diagnosis is gaining an increasing relevance in clinical practice because controlled clinical trials have demonstrated beneficial effects of immunomodulatory therapies [12–14].

Findings in conventional echocardiography are in addition to systolic dysfunction, increase of LV diameter, regional wall motion abnormalities, diastolic dysfunction, and unspecific changes in image texture [15]. However, there is a lack of specific distinguishing features in AMC and suspected DCMi, and in cases where a preserved LV systolic function had been noted, diagnosis of myocarditis has often failed.

The advent of speckle tracking has dramatically expanded the scope of echocardiography. In that, strain and strain rate measurements are new quantitative indices of intrinsic cardiac deformation in real time, providing accurate assessment of regional contractility, and are presumed to be independent of translational motion and other through-plane motion [16–21]. Whether or not these techniques have incremental utility over conventional echocardiography in the course of AMC and DCMi still remains unclear. 

We therefore analysed speckle tracking echocardiography as a new assisting tool for comprehensive assessment of textural and functional alterations of left ventricular myocardium during the course of AMC and DCMi.

## 2. Methods

### 2.1. Study Design

The present study's subjects consisted of 25 consecutive patients (17 males, 8 females) admitted to our institute from January 2007 to November 2010.

The diagnosis of AMC was ascertained clinically. We included patients who presented with a very recent onset of congestive heart failure symptoms, with an abrupt onset of complaints such as angina or dyspnoea, and any infarct-like presentation like elevated serum markers of myocardial injury (troponin T and creatine kinase/creatine kinase MB) and/or newly developed ECG changes (ST segment elevation or T wave inversion). The mean time interval from new onset of symptoms till admission to the hospital was 1.7 ± 0.3 weeks.

The demographic and clinical characteristics of patients at baseline are summarized in Table 1. All patients underwent heart catheterization for the evaluation of the coronary status. After angiographic exclusion of any coronary artery disease and other possible causes of LV dysfunction, EMBs from the right ventricular septum were obtained using a flexible bioptome (Westmed, Germany) via the femoral vein approach [22]. 

All patients were invited to a followup after 6.2 months. Assessment included physical examination, conventional echocardiography, speckle tracking imaging, and EMBs. Patients with severe arterial hypertension, valvular heart disease, and metabolic and endocrine diseases were excluded from the study.

### 2.2. Analysis of Endomyocardial Biopsies

#### 2.2.1. Histology and Immunohistochemical Staining

EMBs were obtained from the RV septum, frozen in liquid nitrogen, and stored at −80°C. For each patient, several biopsies (at least five) were analysed. The histological sections were paraffin-embedded and haematoxylin-eosin-(HE-) stained and examined according to the Dallas criteria [23]. They were analysed by light microscopy for evidence of myocardial necrosis and presence of infiltrates. The coded slides were examined in a blinded fashion. For immunohistological evaluation, specimens were embedded in Tissue Tec (SLEE, Mainz, Germany) and immediately snap-frozen in methyl butane which had been cooled in liquid nitrogen and then again stored at −80°C until processing. Embedded specimens were cut serially into cryosections having a 5 *μ*m thickness and placed on 10% poly-L-lysine precoated slides. Immunohistochemistry was used for the characterization of inflammatory infiltrates and cell adhesion molecules (CAMs). 

Myocardial inflammation was defined by the detection of infiltrating lymphocytes (CD3^+^ T-lymphocytes (Dako, Glostrup, Denmark; dilution 1 : 25) and CD11a/LFA-1^+^ lymphocytes (Immuno Tools, Friesoythe, Germany; dilution 1 : 250), considered as positive intramyocardial inflammation at threshold cell count >14.0 cells/mm^2^) and macrophages (CD11b/Mac-1 (Immuno Tools, Friesoythe, Germany; dilution 1 : 500), considered as positive intramyocardial inflammation at threshold cell count >35.0 cells/mm^2^) in association with enhanced expression of cell adhesion molecules/area fraction (AF); HLA-1 (Dako, Glostrup, Denmark; dilution 1 : 2000), ICAM-1 (Immuno Tools, Friesoythe, Germany; dilution 1 : 800). As secondary antibody, we used the enhancing EnVision peroxidase conjugated anti-mouse antibody (DakoCytomation, Hamburg, Germany). Immunohistological staining was visualized using 3-amino-9-ethylcarbazole (Merck, Darmstadt, Germany) as a chromogenic substrate. Finally, the slides were counterstained in hematoxylin and mounted with Kaiser's gelatinR (Merck, Darmstadt, Germany). The staining and peroxidase reactions in all samples were carried out identically and in parallel for all samples. Immunoreactivity was quantified by digital image analysis (DIA; unit: area fraction) at 200-fold magnification as described elsewhere [24]. Representative images are shown in Figure 3.

#### 2.2.2. Detection of Viral Genomes in EMBs by nPCR and qPCR

Four specimens were submitted for molecular biological investigation of cardiotropic viral genomes according to published techniques [9]. In brief, nested polymerase chain reaction (PCR) was performed on RNA extracted from EMBs for enteroviruses (EVs)—including coxsackie viruses and echoviruses—and in DNA for Epstein-Barr virus (EBV), B19, and human herpes virus 6 (HHV6). As a control for the successful extraction of DNA and RNA from heart muscle tissue, oligonucleotide sequences were chosen from the DNA sequence of the glyceraldehyde 3-phosphate dehydrogenase gene. Specificity of all amplification products was confirmed by automatic DNA sequencing [7]. 

### 2.3. Echocardiographic Analysis

#### 2.3.1. Definition and Assessment of Ventricular Function

Conventional Doppler flow velocities were recorded in the apical 4-chamber view obtained by a Vivid 7 (GE Healthcare, Chalfont St Giles, UK) as described previously [25]. LVEF was measured by using the biplane Simpson's method. The LV mass was calculated according to the formula proposed by Devereux and divided by body surface area for LV mass index calculation [26]. Measurements were performed offline using EchoPAC Version 7.0 (GE, Healthcare, Chalfont, St Giles, UK). Left ventricular dimensions were measured from the standard M-mode using the long parasternal axis view according to the recommendations of the American Society of Echocardiography [27].

#### 2.3.2. Speckle Tracking Imaging

Myocardial deformation measurements were performed using speckle tracking imaging. Three cardiac cycles were recorded as three-beat cine-loop clips in apical four-chamber view and two-chamber view. In each view, a global longitudinal strain and strain rate curve were obtained, including all LV myocardial segments, and using standard ECHOPAC application for two-dimensional strain analysis. The average values of peak systolic longitudinal strain and peak systolic strain rates for all views were calculated as global systolic longitudinal strain (LS) and strain rate (LSR), respectively [28, 29]. Representative images are shown in Figure 4. 

### 2.4. Statistical Methods

Data are shown as median values and standard deviations. After having established that the data were not distributed normally, the nonparametric Mann-Whitney *U* test was used. A probability value of <0.05 was considered statistically significant. Data was analysed with Graphpad 5.01 (PRISM, SanDiego, USA). 

## 3. Results

### 3.1. Demographic and Clinical Data

Baseline population and clinical characteristics are presented in Table 1. The study included 25 patients, 17 males, 8 females, with a mean age of 41.3 ± 12.5 years. The entire cohort had been presented with chest pain, ST elevation, and cardiac biomarker changes and then proceeded to coronary angiography during the same admission. The mean follow-up period was 6.2 months. 

### 3.2. Detection of Viral Genomes, Histology, and Immunohistological Staining in EMBs

At baseline in the acute phase, severe increased immunohistological signs of inflammation, infiltrative cells (mean 57.7 ± 25.6 LFA-1 + lymphocytes/mm² and mean 95.5 ± 44.8 Mac 1 monocytes/macrophages/mm²) as well as cell adhesion molecules (mean 0.082 ± 0.068 HLA-1/AF and 0.051 ± 0.042 ICAM-1/AF) were detected in all patients. All EMB samples were classified as active myocarditis positive according to the Dallas criteria [23]. Therefore, they especially showed infiltrates in a focal pattern in the neighbourhood of myocytolysis in the histology.

At followup in the EMBs of *n* = 17 patients in regard to the immunohistological staining, the number of neither T-lymphocytic nor macrophage infiltrates was increased. Out of these 17 patients, *n* = 8 showed a mild increase of cell adhesion molecules only. However, this, by definition, is not to be considered as intramyocardial inflammation. 

In *n* = 8 patients a persistence of inflammation could be observed. For details see Table 2. The frequencies of viral genomes detected by nPCR are shown in Table 2. The prevalence of virus genomes was not found to be significantly increased in patients with and without persistence of inflammation at the followup.

### 3.3. Heart Dimensions and Systolic Function: Conventional Echocardiography

Conventional echocardiographic measurements at baseline and at follow-up are shown in Table 3. 

Patients in acute myocarditis had markedly decreased LV systolic function (mean LV ejection fraction (LVEF) 40.4 ± 10.3% and fractional shortening (FS) 19.3 ± 4.2%) and ventricular dilation at baseline (left ventricular enddiastolic diameter, LVEDD 59 ± 8 mm). Additionally, an increased septal thickness (13.1 ± 0.4 mm) was observed in *n* = 18 patients with acute myocarditis. The interaction between grade of inflammation and time for fractional shortening was statistically significant (*P* = 0.0005). 

During followup, one patient developed a dilated cardiomyopathy. Six of the patients with an initial mildly reduced EF did not recover fully in regard to their LV function. Twenty of the patients showed a complete normalization of LV function in the followup (Table 3).

At followup, patients for whom no inflammation had been detected showed a marked improvement in the FS (32.1 ± 7.1%), resulting in improved LV systolic function. In the *n* = 8 patients with inflammation in EMB at followup, FS was significantly reduced (21.5 ± 6.0%). Spontaneous resolution of LV thickening was observed in *n* = 14 patients at followup. The left ventricular mass index was significantly different between patients with persistence of inflammation and those with reconvalescence at followup and, moreover, even increased in patients in the acute phase.

Complete normalization of ventricular function was significantly more likely in patients with no inflammation at followup, and the left ventricular diastolic dimension remained significantly smaller in the group of patients with inflammation at followup. One of the patients with an initial mildly reduced LVEF developed DCM. 

### 3.4. Speckle Tracking Imaging

Results of the speckle tracking imaging are presented in Table 3. LSR and LS correlated with LVEF (*r* = 0.573, *P* = 0.03; *r* = 0.05, *P* = 0.02). 

**Figure 1:**
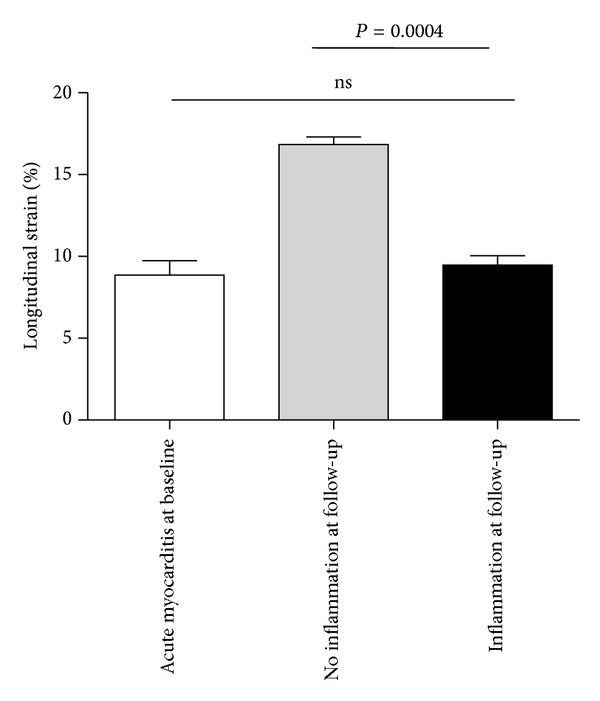
Quantitative analysis of global longitudinal strain (in %) in AMC at baseline and at followup in patients with inflammation (*n* = 8) in EMB and without inflammation (*n* = 17) at followup. Columns represent mean ± standard deviation with *P < 0.05.

**Figure 2:**
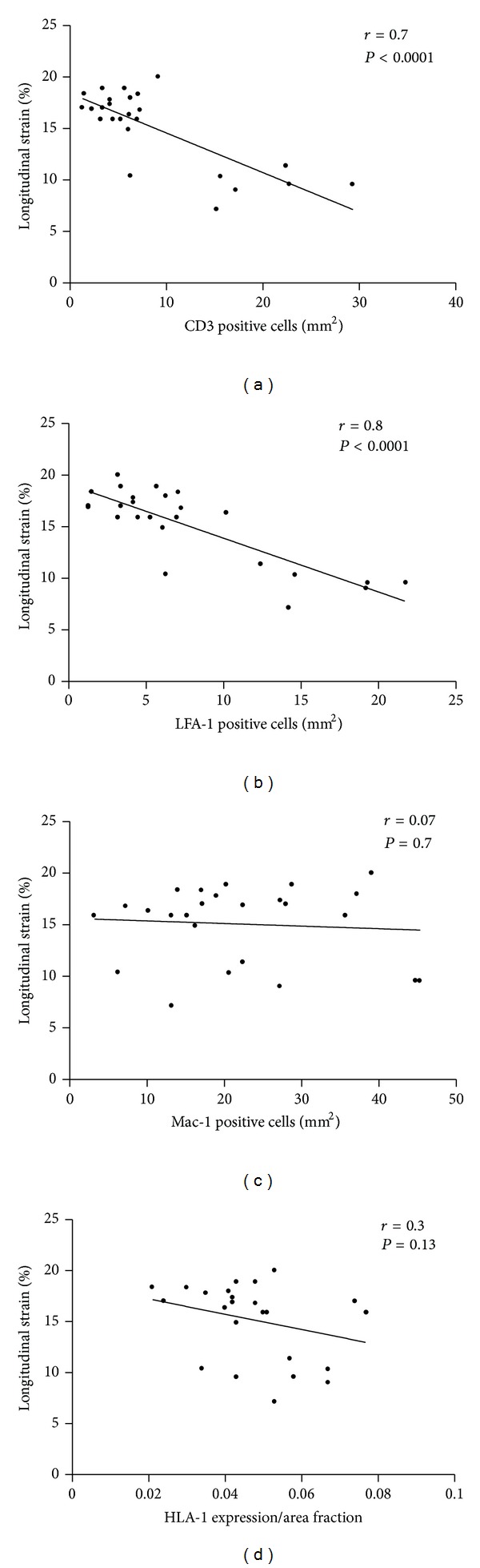
Analysis of global longitudinal strain (in %) correlated with inflammation in EMB. (a) Correlation between global longitudinal strain and CD3 positive T-lymphocytes. (b) Correlation between global longitudinal strain and LFA-1 positive lymphocytes. (c) Correlation between global longitudinal strain and Mac-1 positive cells used as marker for macrophages and (d) correlation between global longitudinal strain and HLA-1 expression.

**Figure 3:**
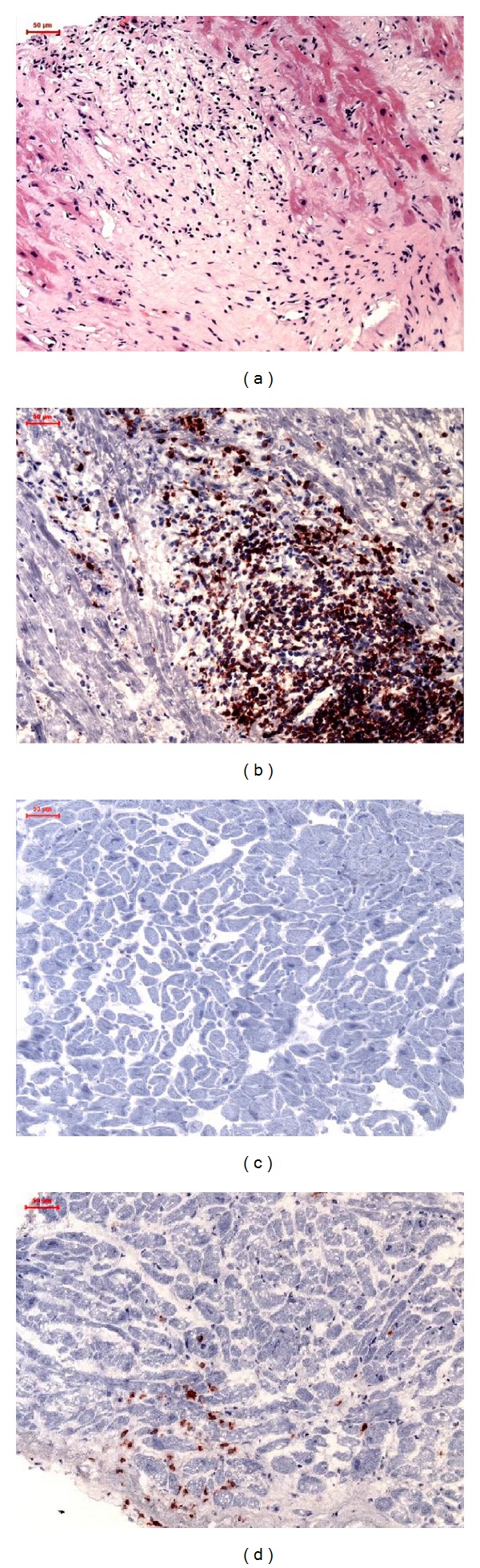
Representative images of EMBs: (a) HE staining of samples from patients with acute myocarditis at baseline, (b) immunohistochemical staining of extremely increased CD3 positive T-lymphocytes in acute myocarditis at baseline, (c) immunohistochemical staining of CD3 positive T-lymphocytes in patients without inflammation at followup, and (d) increased CD3 positive T-lymphocytes in patients with proof of inflammation at followup (magnification 200×).

**Figure 4:**
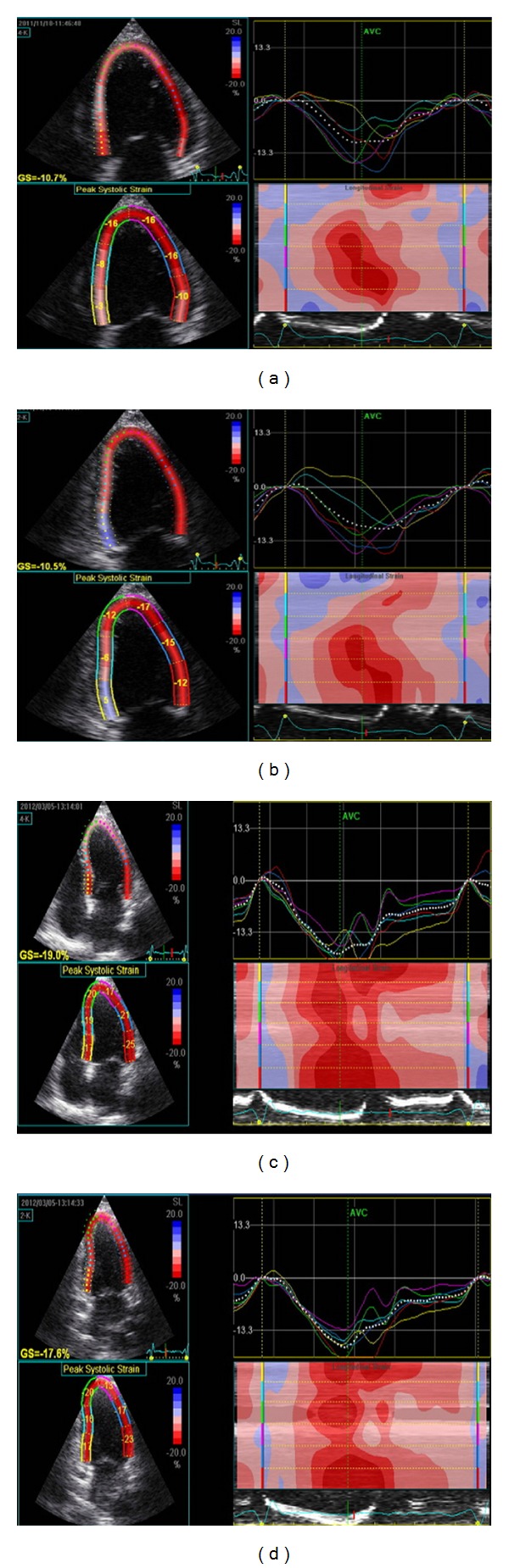
Representative strain imaging by speckle tracking imaging in (a) patients with inflammation in EMB specimens at followup in a 4-chamber view, (b) 2-chamber view, (c) patients without EMB inflammation in a 4-chamber view, (d) 2-chamber view.

In the acute phase all patients showed a reduction in LSR (0.53 ± 0.29 1/s) and LS (−8.36 ± −3.47%) even in those who had preserved LV systolic function (Figure 1). Therefore LS and LSR correlated significantly with grade of inflammation in the acute phase (*P* = 0.001). At follow-up LS and LSR (−9.4 ± −1.4 versus −16.8 ± −2.0%, *P* < 0.0001; 0.78 ± 0.4 versus 1.3 ± 0.3 1/s, resp.) were significantly lower in patients with inflammation, in contrast to the patients without inflammation. In detail, LSR and LS correlate significantly with lymphocytic infiltrates (for CD3 *r* = 0.7, *P* < 0.0001, and LFA-1 *r* = 0.8, *P* < 0.0001) but not with monocytes/macrophages (Mac-1) and CAMs; see Figure 2. No differences in LSR and LS measurements were seen in the acute phase at baseline between the patients who recover and those in whom inflammation was confirmed in the followup.

## 4. Discussion

The salient finding of this study is that speckle tracking imaging is an assisting tool for comprehensive assessment of textural and functional alterations of left ventricular myocardium and correlates with EMB-proven inflammation in patients with acute myocarditis and inflammatory cardiomyopathies. In our study we could show wall motion abnormalities demonstrated by decreased longitudinal strain in patients with EMB-proven intramyocardial inflammation in DCMi even in those who had a preserved LV systolic function.

Diagnosing myocarditis and inflammatory cardiomyopathy is difficult since the symptoms are characterized by a pronounced variability. Previous studies of conventional echocardiography in myocarditis have demonstrated a variety of echocardiographic findings, and in addition to systolic dysfunction, regional wall abnormalities, changes in image texture, and pericardial effusion had been reported [19, 30–32]. However, until now, no study has been reported which attempts to distinguish the spectrum of EMB-proven intramyocardial processes. They are mainly used to rule out other causes of heart failure. Our study confirms that patients with AMC have a decreased longitudinal strain, and, in addition, patients with DCMi have a speckle tracking imaging presentation that is distinct from that of patients without intramyocardial inflammation proved by EMBs. Beyond that, we discriminate in AMC and DCMi patients LS and LSR and septal textural alteration with wall dispersion recognized by conventional echocardiography and correlating with EMB-proven inflammation. We noted in *n* = 18 patients transient LV hypertrophy in AMC. Spontaneous resolution of LV thickening was observed in 14 patients at followup. At the six-month followup, in these patients, a significant improvement in LVEF could be observed. Here, we are in line with previous studies involving observed transient LV hypertrophy in the setting of acute myocarditis [33–36]. The presence of myocardial interstitial oedema leads to a thickening of the ventricular wall in acute myocarditis. The increased thickness is likely due to a great inflammatory response seen in EMBs. However, adhesion molecules, expressed on endothelial cells and involved in process of increased vascular leakage, did not correlate with LS or LSR. 

Moreover, interstitial edema may contribute to both thickened ventricular walls and decreased ventricular contractility in this disorder. The endocardial layer, among the different intramural layers of LV wall, is that which better contributes to the overall myocardial thickening. Intramyocardial infiltration can be located in the epicardial layer of ventricular wall during myocarditis but this cannot be associated with any clear evidence of wall motion abnormalities. Therefore, the conventional transthoracic echocardiography cannot identify the presence of a segmental myocardial dysfunction. Di Bella et al. reported that strain echocardiography was able to identify longitudinal segmental myocardial dysfunction derived from edema in the acute phase of myocarditis [29, 37]. 

Additionally, in previous studies a significant relationship between the left ventricular mass indexes during acute myocarditis was observed [24]. Elevation of the left ventricular mass index during the acute phase myocarditis is due to myocardial edema from increased infiltration and vascular permeability. Therefore, it is presumed that the decreased longitudinal strain in this study was the result of myocardial wall motion disturbance from the elevation of the left ventricular mass index. Also, decreased longitudinal strain should be regarded as a myocardial functional disturbance in the myocarditis because elevation of the left ventricular mass index is an index of geometric change in inflammatory cardiomyopathy.

Speckle tracking imaging is a promising and noninvasive method to help identify intramyocardial inflammation even in the case of lack of wall motion abnormalities. Systolic strain rate is usually regarded as the most significant index of left ventricular contractility among the indices obtained from myocardial deformation analyses having been described in patients with other forms of cardiomyopathy (e.g., ischemic or hypertrophic cardiomyopathy). 

In our study, at followup LS and LSR were significantly lower in patients with inflammation in contrast to those patients without inflammation. In detail, LSR and LS correlate significantly with lymphocytic infiltrates which is the strongest predictor of poor outcome [11]. Here, we reported on the use of strain echocardiography to identify longitudinal myocardial dysfunction derived from infiltration. Decreased left ventricular FS has been shown to result from the decreased systolic myocardial contractility, caused by myocarditis, during the acute phase. We observed the same finding during the acute phase of the disease with speckle tracking echocardiography. 

The acute phase of myocarditis is characterized by extensive cellular infiltration, especially in a focal pattern and necrosis, whereas in the case of chronic myocarditis, no myocyte necrosis apart from cellular infiltration could be detected. Whether speckle tracking imaging is able to recognize focal infiltrative pattern or deformation associated with edema remains unclear. In spontaneous course of the disease the acute phase is necessary for virus elimination. Thus, virus genomes cannot be detected in a number of cases in the acute phase. In regard to presence of viruses until now none of our echocardiographic findings seem to be able to detect viral genomes, and this has significant therapeutic implications. Therefore, an unequivocally confirmed EMB diagnosis is the crucial prerequisite for differential diagnostic evaluation and the specific treatment strategies derived from this.

Nevertheless, in our study LS and LSR are impaired in EMB-dependent DCMi having diffuse intramyocardial cellular lymphocytic infiltrative pattern and should therefore warrant a useful adjunctive investigation.

## Figures and Tables

**Table 1:** Clinical characteristics.

Parameter	Acute myocarditis at baseline	Patients without inflammation at followup	Patients with inflammation at followup
Gender (m/f), *n*	17/8	11/6	5/3
Age, y	41.3 ± 12.5	40.4 ± 11.5	43.5 ± 14.7
BMI, kg/m²	27.0 ± 3.3	26.7 ± 3.5	27.1 ± 3.4
Arrhythmias, *n*	8	1	4
(i) Atrial fibrillation/flutter	6	1	2
(ii) Ventricular tachycardia	1	0	0
(iii) Bradycardia	0	0	0
Atrial ventricular block, *n*	0	0	0
Left bundle branch block, *n*	0	0	0
Hypertension, *n*	7	5	2
Hyperlipidaemia, *n*	4	2	2
Diabetes mellitus, *n*	1	1	0
*β*-blockers, *n*	19	8	4
ACE inhibitors, *n*	21	4	4
Statins, *n*	4	2	2

Data were not significant between patients with and without inflammation at followup. Data are shown as mean ± standard deviation.

BMI: body mass index; ACE: angiotensin converting enzyme.

**Table 2:** Intramyocardial inflammation by immunohistochemistry and virus genomes by PCR.

Parameter	Acute myocarditis at baseline	Patients without Inflammation at followup	Patients with Inflammation at followup
Inflammation			
CD3^+^/mm^2^	45.0 ± 17.0	4.9 ± 2.1	20.5 ± 5.4*
LFA-1^+^/mm^2^	57.7 ± 25.6	3.3 ± 7.1	29.3 ± 7.1*
Mac-1^+^/mm^2^	95.5 ± 44.8	19.8 ± 10.4	28.8 ± 13.2
HLA class I/AF	0.082 ± 0.068	0.045 ± 0.015	0.057 ± 0.009
ICAM-1/AF	0.051 ± 0.042	0.023 ± 0.017	0.033 ± 0.0137
Virus			
Enterovirus, *n*	2	0	1
B19, *n*	17	9	10
HHV-6, *n*	1	1	0

*Significant differences between patients with and without inflammation at followup. ns: not significant. Data are shown as mean ± standard deviation.

**Table 3:** Conventional echocardiographic parameters and speckle tracking echocardiography at baseline and at followup.

Parameter	Acute myocarditis at baseline	Patients without inflammation at followup	Patients with inflammation at followup
LVEF, %	40.4 ± 10.3	65.3 ± 6.1	56.2 ± 7.2*
Regional wall abnormalities, *n*	10	2	2
LVEDD, mm	59 ± 8	51 ± 3	55 ± 4
LVESD, mm	51 ± 7	39 ± 5	42 ± 7
LA, mL	35.4 ± 5.4	34.6 ± 4.2	37.8 ± 6.3
Septum, mm	13.1 ± 0.4	10.4 ± 0.5	11.5 ± 1.4
Posterior wall, mm	11.2 ± 1.7	11.2 ± 0.5	10.2 ± 0.8
Dispersing wall, *n*	16	3	3
LV mass index, g/m²	91.1 ± 18.4	76.3 ± 10.1	86.2 ± 11.1*
FS, %	19.3 ± 4.2	32.1 ± 7.1	21.5 ± 6.0*
LSR, 1/s	0.53 ± 0.29	1.3 ± 0.3	0.78 ± 0.4*
LS, %	8.36 ± 3.47	16.87 ± 2.0	9.4 ± 1.4*
LSRsep, 1/s	0.54 ± 0.34	0.81 ± 0.41	0.51 ± 0.14*
LSsep, %	12.98 ± 2.76	16.65 ± 3.98	13.43 ± 4.32*

*Significant differences between patients with and without inflammation at followup. ns: not significant. Data are shown as mean ± standard deviation.

LVEDD: left ventricular enddiastolic diameter, LVESD: left ventricular endsystolic diameter, LA: left atrium, FS: fractional shortening, LSR: global systolic longitudinal strain rate, LSRsep: septal longitudinal systolic strain rate, LS: global systolic longitudinal strain, LSsep: septal systolic longitudinal strain.

## Abbreviations

AMC: Acute myocarditis
AF: Area fraction
CAMs: Cell adhesion molecules
DCMi: Inflammatory cardiomyopathy
DIA: Digital image analysis
EF: Ejection fraction
EMB: Endomyocardial biopsy
FS: Fractional shortening
HLA-1: Human leukocyte antigen-1
ICAM-1: Intercellular cell adhesion molecule-1
LFA-1: Leukocyte function antigen-1
LS: Longitudinal strain
LSR: Longitudinal systolic strain rate
LSR sep: Septal longitudinal systolic strain rate
LV: Left ventricular
LVEDD: Left ventricular end diastolic diameter
LVESD: Left ventricular end systolic diameter
PCR: Polymerase chain reaction
VCAM-1: Vascular cell adhesion molecule-1.
